# Development of a standardized single‐session cardiopulmonary exercise test for combined assessment of peak oxygen uptake and on/off‐kinetics

**DOI:** 10.1113/EP092337

**Published:** 2025-03-20

**Authors:** Jefferson L. Santana, Till Enzner, Britney Blunderfield, Asher A. Mendelson, Rodrigo Villar

**Affiliations:** ^1^ Cardiorespiratory & Physiology of Exercise Research Laboratory, Faculty of Kinesiology and Recreation Management University of Manitoba Winnipeg Canada; ^2^ Department of Physiology and Pathophysiology, Rady Faculty of Health Sciences University of Manitoba Winnipeg Canada; ^3^ Section of Critical Care Medicine, Department of Internal Medicine, Rady Faculty of Health Sciences University of Manitoba Winnipeg Canada

**Keywords:** cardiopulmonary exercise test (CPET), constant work rate (CWR), CPET feasibility, on/off‐kinetics, peak oxygen uptake

## Abstract

Peak oxygen uptake (${\dot{V}}_{O_{2}\mathrm{peak}}$) and ${\dot{V}}_{O_{2}}$ on/off‐kinetics are key indicators of exercise capacity and health outcomes, but their assessment often requires separate laboratory visits, which limits feasibility. This cross‐sectional study aimed to develop a single cardiopulmonary exercise test (CPET) for both assessments. We designed a single‐session combined CPET protocol using an upright cycle ergometer in healthy volunteers (*n *= 20). ${\dot{V}}_{O_{2}\mathrm{peak}}$ was first estimated using an a priori formula. The constant work rate (CWR) part of the test (on‐kinetics) was set to an intensity of 30% ${\dot{V}}_{O_{2}\mathrm{reserve}}$. After an incremental test to measure ${\dot{V}}_{O_{2}\mathrm{peak}}$, a 10‐min recovery period was used to evaluate off‐kinetics. Twenty volunteers (9 females and 11 males), 28.0 ± 8.1 years completed the protocol. No significant differences were found between predicted and measured ${\dot{V}}_{O_{2}\mathrm{peak}}$ (*P *= 0.47). A strong correlation (*r *= 0.88) and good agreement (Bland–Altman bias = −0.82 mL kg^−1^ min^−1^) were found between the calculated/actual individuals’ 30% ${\dot{V}}_{O_{2}\mathrm{reserve}}$ (mL kg^−1^ min^−1^) and the measured steady‐state ${\dot{V}}_{O_{2}}$ at CWR. The measured exercise intensity at CWR closely matched the target of 30%, with no statistical differences, with an average difference of 0.2 percentage points. Small–medium Cohen's *d* (0.16) indicated high similarity between predicted and measured ${\dot{V}}_{O_{2}\mathrm{peak}}$. ${\dot{V}}_{O_{2}}$ on‐ and off‐kinetics analyses were also performed for all participants with mono‐exponential fittings. A single‐session protocol for the combined assessment of ${\dot{V}}_{O_{2}\mathrm{peak}}$ and ${\dot{V}}_{O_{2}}$ on/off‐kinetics was developed. This protocol will enable greater recruitment and participation in research and enhanced detail for clinical CPET use. Future research should evaluate intra‐ and inter‐participant reproducibility over repeated sessions.

## Abstract (highlights)

- **What is the central question of this study?**
  Peak oxygen uptake (${\dot{V}}_{O_{2}\mathrm{peak}}$) and ${\dot{V}}_{O_{2}}$ on/off‐kinetics are key indicators of exercise capacity and health outcomes, but their assessment often requires separate laboratory visits: can a single cardiopulmonary exercise test (CPET) for both assessments be developed?
- **What is the main finding and its importance?**
  A single‐session protocol for the combined assessment of ${\dot{V}}_{O_{2}\mathrm{peak}}$ and ${\dot{V}}_{O_{2}}$ on/off‐kinetics was developed. This protocol will enable greater recruitment and participation in research and enhanced detail for clinical CPET use.

## 1 INTRODUCTION

The cardiopulmonary exercise test (CPET) is a non‐invasive method for assessing aerobic power, predicting health outcomes, facilitating diagnosis of cardiopulmonary diseases, and evaluating interventions in clinical and research settings (Dallaire et al., 2017; Wasserman et al., 2012; Weisman et al., 2003). Maximal CPET yields insights into cardiovascular, ventilatory, pulmonary and systemic influences on exercise tolerance and is a robust prognostic factor for many health conditions (Goulding et al., 2021; Wasserman et al., 2012). However, activities of daily living are typically performed at submaximal levels (i.e., frequent rest to light/moderate intensity transitions), evoking different physiological mechanisms from those elicited by maximal CPET to determine peak oxygen uptake (${\dot{V}}_{O_{2}\mathrm{peak}}$) (Jones & Poole, 2005; Longobardi et al., 2022).

The significance of obtaining data related to ${\dot{V}}_{O_{2}}$ kinetics at submaximal levels has been acknowledged in recent decades (Burnley et al., 2011; Grassi et al., 2011; Hughson, 2009). Described as the rate of oxidative phosphorylation adjustment to a sudden increase in energy demand, ${\dot{V}}_{O_{2}}$ on/off‐kinetics plays a crucial role in understanding the interplay between various physiological mechanisms (Grassi et al., 2011). Kinetic analysis contributes to a deeper understanding of exercise capacity, fitness, quality of life and survival at a physiological level (Goulding et al., 2021). The understanding of ${\dot{V}}_{O_{2}}$ on/off‐kinetics is important for assessing exercise efficiency (Xu & Rhodes, 1999) and may play a role in the early detection of abnormalities, quantifying aerobic fitness, which is associated with exercise tolerance, and effectively monitoring treatment (Dallaire et al., 2017; Wasserman et al., 2012; Weisman et al., 2003). The ${\dot{V}}_{O_{2}}$ kinetics is intricately associated with oxidative phosphorylation and metabolic responses to exercise (Goulding et al., 2021; Grassi et al., 2011).

Currently for research purposes, obtaining information regarding ${\dot{V}}_{O_{2}\mathrm{peak}}$ and ${\dot{V}}_{O_{2}}$ on/off‐kinetics requires a minimum of two separate visits of around 1 h for the first and 2.5 h for the second. This is problematic as it significantly impacts recruitment and retention, considered one of the most challenging aspects of clinical research (McDonald et al., 2006). Longitudinal (multiple visits) studies are particularly susceptible to missing data (Okpara et al., 2022), with only 40% of randomized controlled trials (RCTs) meeting recruitment targets and 32% extending their recruitment periods (Walters et al., 2017). This information highlights the low retention rates and time burden with the multiple visits design. Challenges are even more pronounced in research investigating high‐risk populations, requiring adaptations to accommodate their limitations and current needs (Saulnier et al., 2022). In clinical practice, because of limitations to healthcare resources and time constraints for patients, ${\dot{V}}_{O_{2}}$ on/off‐kinetics are rarely evaluated and do not comprise routine standard of care for clinical CPET (Weisman et al., 2003).

Integrating ${\dot{V}}_{O_{2}\mathrm{peak}}$ and ${\dot{V}}_{O_{2}}$ on/off‐kinetics, within a single session presents methodological challenges. Typically, exercise intensity at a constant work rate (CWR) for on/off‐kinetics is derived based on a known ${\dot{V}}_{O_{2}\mathrm{peak}}$ or ventilatory threshold from a prior CPET session (George et al., 2018; Hughson & Morrissey, 1982; Murias et al., 2011b). Although many methods exist, there is no universally accepted method for estimating ${\dot{V}}_{O_{2}\mathrm{peak}}$ to derive CWR exercise without prior CPET. This raises concerns about the direct comparability of ${\dot{V}}_{O_{2}}$ on‐kinetics between participants for a fixed CWR, as this may reflect different relative intensities of work for each participant. A recent study by Longobardi et al. (2022) used a single protocol session integrating ${\dot{V}}_{O_{2}\mathrm{peak}}$ and ${\dot{V}}_{O_{2}}$ on/off‐kinetics. However, the work rate (exercise intensity) selected for the steady‐state phase of the protocol (rest to exercise transition) was determined based on participants' subjective perception and the duration of the CWR (3 min) may be too short for attaining steady‐state ${\dot{V}}_{O_{2}}$ for some participants. A single‐session testing protocol for ${\dot{V}}_{O_{2}\mathrm{peak}}$ and standardized relative CWR for on/off‐kinetics would enhance the efficiency of research practices and diminish barriers to healthcare access, all of which reduce vulnerable populations slipping between the cracks of our healthcare system. It will also offer a more patient‐centred and streamlined approach to assess exercise capacity, predict health outcomes, facilitate diagnosis and evaluate treatment effectiveness.

To address the aforementioned challenges, our study aims to develop a single CPET protocol for simultaneous determination of ${\dot{V}}_{O_{2}\mathrm{peak}}$ and ${\dot{V}}_{O_{2}}$ on/off‐kinetics. This protocol was designed to minimize barriers to recruitment and retention while providing comparable relative intensity between participants during the CWR exercise phase. We hypothesize that the proposed experimental tests will permit ${\dot{V}}_{O_{2}\mathrm{peak}}$ and ${\dot{V}}_{O_{2}}$ on/off‐kinetics to be incorporated into a single protocol.

## 2 METHODS

### 2.1 Ethical approval

This study was reviewed and approved by the University of Manitoba Health Research Ethics Board (Ethics approval protocol number HE2022‐0058) and was in agreement with the *Declaration of Helsinki*.

### 2.2 Study design

A CPET was employed to determine the ${\dot{V}}_{O_{2}\mathrm{peak}}$ and ${\dot{V}}_{O_{2}}$ on/off‐kinetics, using an observational cross‐section design following the Strengthening the Reporting of Observational Studies in Epidemiology (STROBE) guidelines. Recruitment, exposure and data collection took place from July to October 2023. Data were collected during a single visit on a stationary cycle ergometer (Lode Corival, Lode B.V. Medical Technology, Groningen, Netherlands).

### 2.3 Participants

Convenient and snowball sampling methods (Biernacki & Waldorf, 1981) were used to recruit participants. Twenty healthy adults (9 females and 11 males) volunteered for this study. Individuals of both sexes (18+ years), non‐smokers, and non‐pregnant were included in this study. Exclusion criteria included orthopaedic complications, smoking habits, experienced cardiac/lung diseases (e.g., heart failure, emphysema), and long‐distance athletes (e.g., runners, cyclists, triathletes), which were considered outside of the normative healthy and moderately physically active individual. All participants were given detailed verbal and written information about the experimental procedures and potential risks involved before signing an informed consent form approved by the Research Ethics Board. It was requested for all participants to refrain from consuming alcohol and engaging in vigorous exercise for 24 h before testing, and from consuming a large meal 2 h before testing.

### 2.4 Experimental protocols

Before CPET testing, participants’ demographic information was collected and they completed the Get Active Questionnaire (GAC) as recommended by the Canadian Society for Exercise Physiology (CSEP) (Canadian Society for Exercise Physiology, 2021) to identify any potential restrictions on practicing exercise. Also, resting systolic blood pressure (SBP_rest_) diastolic blood pressure (DBP_rest_) and resting heart rate (HR_rest_) were measured following CSEP guidelines (Canadian Society for Exercise Physiology, 2021) before exercise. All participants reported values below the safety cut‐offs (blood pressure ≤140/90 mmHg; heart rate (HR) ≤100 beats min^−1^) (Headley, 2018). The Paffenberger Physical Activity Questionnaire (Paffenbarger et al., 1993) was used to estimate the active level of the participants (i.e., energy expenditure (EE) per week). After these initial safety assessments, height, body mass and body mass index (BMI) were assessed. Then, participants were fitted with an air‐cushioned mask, allowing ventilatory measurements.

**FIGURE 1:**
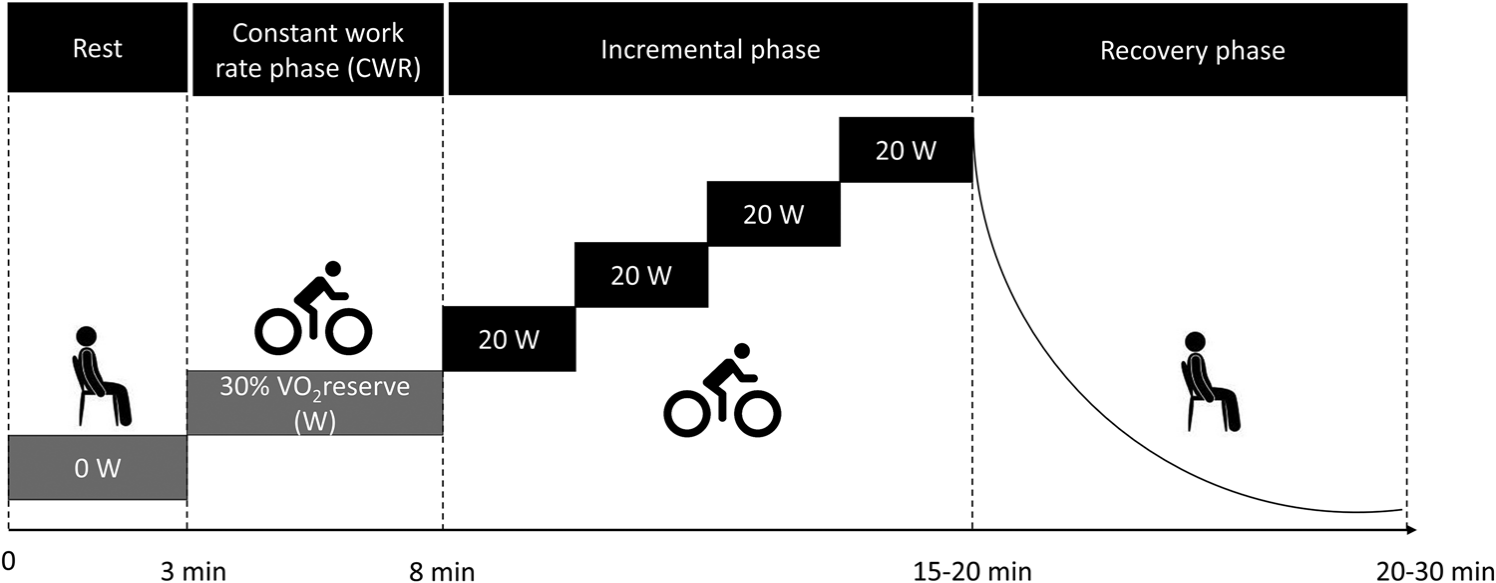
Illustration of the incremental cardiopulmonary exercise test (CPET) protocol.

A diagram of the experiment protocol can be found in Figure 1. The CPET protocol consists of a baseline resting ${\dot{V}}_{O_{2}}$ measurement (mean of 6 readings with 30 s intervals) while seated in a chair. This resting ${\dot{V}}_{O_{2}}$ measurement was used to estimate the load (W) for the CWR phase (see details below) and was manually programmed into the cycle ergometer. Following this, the participants were requested to move and sit on the cycle ergometer; when they were accommodated and feeling comfortable on the bike, a 3 min baseline at rest, with no pedaling, was recorded. At the end of the baseline, we orally provided the command for them to start cycling. The participants then underwent a 5 min steady‐state exercise at an intensity of 30% of their estimated ${\dot{V}}_{O_{2}\mathrm{reserve}}$ with a cadence of 60 rpm. Following the CWR phase, progressive resistance increments of 20 W min^−1^ until volitional exhaustion were implemented. Termination criteria included voluntary exhaustion or inability to maintain the required cadence (<50 rpm) for half stage. Participants were also instructed to refrain from speaking during the protocol to minimize data artifacts. The CPET concluded with a 10‐min recovery period. To address potential sources of bias, we tried to maintain the same level of motivational encouragement toward each participant during the incremental phase of the protocol by using words such as ‘c'mon!’, ‘keep going!’, ‘you're doing great!’ and ‘you can do it!’.

The CPET was conducted in a quiet room (Cardiorespiratory and Physiology of Exercise Research Laboratory) with a controlled temperature of 21.0 ± 1.0°C, and humidity and barometric pressure at ambient.

### 2.5 Estimation of the work rate to determine ${\dot{V}}_{O_{2}}$ on‐kinetics dynamic response

To date, there are no commonly established methods for defining a comparable relative work rate between participants during exercise at a CWR without prior testing to determine individuals ${\dot{V}}_{O_{2}\mathrm{peak}}$, making the direct comparability of ${\dot{V}}_{O_{2}}$ on‐kinetics between participants a major issue. Therefore, a key step to successfully implementing a single‐session CPET protocol for combined assessment of ${\dot{V}}_{O_{2}\mathrm{peak}}$ and ${\dot{V}}_{O_{2}}$ on/off‐kinetics is the accurate estimation of ${\dot{V}}_{O_{2}\mathrm{peak}}$. This ensures that the CWR exercise is set at a similar relative intensity, allowing for meaningful comparisons between participants.

To do so, we proposed an approach with four steps to standardize and determine the CWR exercise intensity during this CPET protocol, as described below:

1. Prediction of ${\dot{V}}_{O_{2}\mathrm{peak}}$. The Hansen–Wassermann equations (Wasserman et al., 2012) recommended by the American Thoracic Society (ATS) and the American College of Chest Physicians (ACCP) committee (Weisman et al., 2003) were used to predict ${\dot{V}}_{O_{2}\mathrm{peak}}$ (L min^−1^). Briefly, the equations used for females and males are, respectively:
(1) $${\dot{V}}_{O_{2}\mathrm{peak}}\left(\mathrm{Females}\right)=0.001\times H\times \left(14.783-0.11\times A\right)+0.006\times \mathrm{Fdw}$$

where H(cm) and A(years) are height and age, respectively, and Fdw is the difference between the actual and the ideal weight (kg) for females, which is considered: 0.65×H−42.8.

(2) $${\dot{V}}_{O_{2}\mathrm{peak}}\left(\mathrm{Males}\right)=0.0337\times H-0.000165\times A\times H-1.963+F\times \mathrm{Mdw}$$

where Mdw is the difference between the actual and the ideal weight (kg) for males, which is considered 0.79×H−60.7; the term F is a correction factor, for cases where the actual weight equals or exceeds the ideal weight: F=0.006, otherwise, F=0.014.

2. Calculation of ${\dot{V}}_{O_{2}\mathrm{reserve}}$. ${\dot{V}}_{O_{2}\mathrm{reserve}}$ was calculated as the difference between the estimated ${\dot{V}}_{O_{2}\mathrm{peak}}$ from step 1 and measured ${\dot{V}}_{O_{2}}$ at rest (${\dot{V}}_{O_{2}\mathrm{rest}}$), as previously described.
3. Determination of target ${\dot{V}}_{O_{2}\mathrm{reserve}}$. The target ${\dot{V}}_{O_{2}\mathrm{reserve}}$ was determined using Equation (3) below, where the ${\dot{V}}_{O_{2}\mathrm{reserve}}$ of the individual (step 2) is multiplied by the desired intensity and then added to the measured ${\dot{V}}_{O_{2}}$ at rest:
(3) $$\begin{array}{ccc} \mathrm{Target}\;{\dot{V}}_{O_{2}\mathrm{reserve}} & = & \left[\left({\dot{V}}_{O_{2}\mathrm{peak}}-{\dot{V}}_{O_{2}\mathrm{rest}}\right)\times \left(\%\;\mathrm{desired}\;\mathrm{intensity}\right)\right]\\ & & +{\dot{V}}_{O_{2}\mathrm{rest}} \end{array}$$

where ${\dot{V}}_{O_{2}\mathrm{peak}}$ $\left(\mathrm{mL}\;\;kg^{-1}\;\mathrm{mi}n^{-1}\right)$ is the value estimated in step 1 but relative to body mass; ${\dot{V}}_{O_{2}\mathrm{rest}}$ $\left(\mathrm{mL}\;\;kg^{-1}\;\mathrm{mi}n^{-1}\right)$ is the value of oxygen uptake at rest, and percentage desired intensity (decimal form) is the desired intensity relative to the individual's ${\dot{V}}_{O_{2}\mathrm{reserve}}$ to perform the exercise. The selected intensity of 30% ${\dot{V}}_{O_{2}\mathrm{reserve}}$, a light intensity according to The American College of Sports Medicine (ACSM) (Garber, 2018), aimed to maximize the chance that the participants would perform the CWR exercise phase below the lactate threshold, reach a steady state (SS) ${\dot{V}}_{O_{2}}$ in 5 min, and at the same time interfere as little as possible in the following progressive incremental phase of the protocol.

4. Estimation of work rate in watts. ACSM provides metabolic calculations to estimate the work rate (i.e., power) for common physical activities, such as leg cycling, using (Garber, 2018):
(4) $$\mathrm{Work}\;\mathrm{rate}\;\left[W\right]=\frac{\mathrm{body}\;\mathrm{mass}\;\times \;\left({\dot{V}}_{O_{2}}-7\right)}{1.8}\;\times 0.164$$

where the body mass of the individual is expressed in kg and the ${\dot{V}}_{O_{2}}$ ($\mathrm{mL}\;\;kg^{-1}\;\mathrm{mi}n^{-1}$) is the target ${\dot{V}}_{O_{2}\mathrm{reserve}}$ (Equation (3)). Thus, Equation (4) determines the required work rate for the CWR phase during the proposed CPET protocol. This metabolic calculation is most accurate for work rates between 50 and 200 W (Garber, 2018), which is the case in this study.

### 2.6 Data acquisition

Pulmonary oxygen uptake (${\dot{V}}_{O_{2}}$) and carbon dioxide output (${\dot{V}}_{CO_{2}}$) were recorded and monitored using the Vyntus CPX Metabolic cart (Vyaire Medical, Mettawa, IL, USA). A bidirectional breath‐by‐breath volume sensor (DVT Vyntus CPX, Vyaire Medical) and an air‐cushioned mask were used to collect the ventilatory variables. Calibration was done before each test, using the Vyaire SentrySuite software platform on the Vyntus, which provides automatic calibration and verification procedures.

### 2.7 Data analysis

Initially, the preprocessing of ${\dot{V}}_{O_{2}}$ data was performed to remove outliers (≥3 standard deviation (SD) of local mean) using a sliding window of five points. The signals were then linearly interpolated and resampled to 1 Hz, and time‐aligned with zero matching the onset of exercise. Following this, on/off‐kinetics analysis was performed using a mono‐exponential model to fit the pulmonary or primary component of the ${\dot{V}}_{O_{2}}$ response (i.e., phase II) by skipping the cardiogenic phase (i.e., phase I), fixed at 20 s in length, as previously recommended (Hughson, 2009; Murias et al., 2011a). The exponential models are characterized by the parameters tau (τ), which is the time constant of the exponential function, representing the time taken to reach 63% of the SS or final value; the time delay (TD) taken to the exponential response/behaviour start (such that the model is not constrained to pass through the origin); and *A*_0_ and *A*_1_, which represent the baseline value (initial ${\dot{V}}_{O_{2}}$ values) and the amplitude reached from baseline to steady‐state ${\dot{V}}_{O_{2}}$ (i.e., SS ${\dot{V}}_{O_{2}}$ − baseline value). The quality of the fitting was assured by the analysis of residuals, the degree of linear correlation between the experimental data and fitted function (*r*), the 95% confidence interval band (CI_95_), and the significance level (*P*‐value) of the estimated parameters. The SS ${\dot{V}}_{O_{2}}$ was calculated as the average of the last 2 min of the CWR exercise phase. The ${\dot{V}}_{O_{2}\mathrm{peak}}$ was estimated by taking the highest value of the last 30 s before volitional exhaustion after applying a 20‐s moving average (Hedge & Hughson, 2020; Robergs et al., 2010). All the pre‐processing and fitting analyses were performed using MATLAB coding (MATLAB R2022b, MathWorks, Inc., Natick, MA, USA) and Labview (LabView v20.0.1, National Instruments Corp, Austin, TX, USA).

### 2.8 Statistical analysis

Descriptive data were expressed as the mean ± SD or the mean ± 95% confidence interval (CI_95_). The normal distribution of the data was tested using the Shapiro–Wilk normality test and equal variance using the Brown–Forsythe equal variance test. The statistical analysis for comparisons between predicted and measured variables was determined with (i) percentage differences between predicted and measured variables for general weighted inspection of the difference; (ii) Student's paired *t*‐test to identify statistically significant differences between predicted and measured variables; (iii) linear regression/correlational analysis with measured variables as the dependent and predicted as the independent variable; (iv) Bland–Altman analysis to assess the agreement between predicted and measured variables; and (v) Cohens’ *d* test in order to assess the size of the difference/similarity between predicted and measured variables. The Pearson product‐moment correlation coefficient (*r*) was considered very strong (*r* = 0.90–1.00), strong (*r* = 0.70–0.89), moderate (*r* = 0.40–0.69), weak (*r* = 0.10–0.39) and negligible (*r* = 0.00–0.10) (Overholser & Sowinski, 2008; Schober & Schwarte, 2018). JASP Team (2023) (Version 0.17.2.1, JASP, Amsterdam, Netherlands) was used for statistical analysis and GraphPad Prism 8 (Version 8.4.2, GraphPad Software, Boston, MA, USA) for plotting and data representation purposes. Significance was set at *P* < 0.05.

## 3 RESULTS

The overall group (*n* = 20) consisted of moderately active healthy female (*n *= 9) and male (*n *= 11) adults. Participants’ characteristics regarding age, height, body mass, BMI, resting heart rate (HR_rest_), resting systolic blood pressure (SBP_rest_), resting diastolic blood pressure (DBP_rest_), resting ${\dot{V}}_{O_{2}}$ (${\dot{V}}_{O_{2}\mathrm{rest}}$), and weekly energy expenditure (EE) are displayed in Table 1. Sex‐disaggregated data are provided, but comparative statistical analysis between sexes was not conducted, as it was not the primary focus of this proof‐of‐concept study.

**TABLE 1:** Sample characteristics divided into female, male and combined groups.

Characteristic	Female (*n *= 9)	Male (*n *= 11)	Combined (*n *= 20)
Age (years)	27.8 ± 8.7	28.2 ± 8.0	28.0 ± 8.1
Height (cm)	167.0 ± 7.3	175.0 ± 5.5	171.4 ± 7.4
Body mass (kg)	61.6 ± 8.3	75.9 ± 10.2	69.5 ± 11.7
BMI (kg m^−2^)	22.2 ± 3.2	25.0 ± 3.0	23.7 ± 3.3
HR_rest_ (beats min^−1^)	73 ± 12	73 ± 9	73 ± 10
SBP_rest_ (mmHg)	109 ± 10	131 ± 9	121 ± 14
DBP_rest_ (mmHg)	69 ± 10	76 ± 9	73 ± 10
${\dot{V}}_{O_{2}\mathrm{rest}}$ (mL kg^−1^ min^−1^)	3.5 ± 0.8	3.8 ± 0.7	3.7 ± 0.8
EE (kcal week^−1^)	6446 ± 3002	6903 ± 3193	6697 ± 3036

*Note*: Values are means ± SD. HR_rest_, resting heart rate; SBP_rest_, resting systolic blood pressure; DBP_rest_, resting diastolic blood pressure; ${\dot{V}}_{O_{2}\mathrm{rest}}$ = resting oxygen consumption; EE, energy expenditure.

**FIGURE 2:**
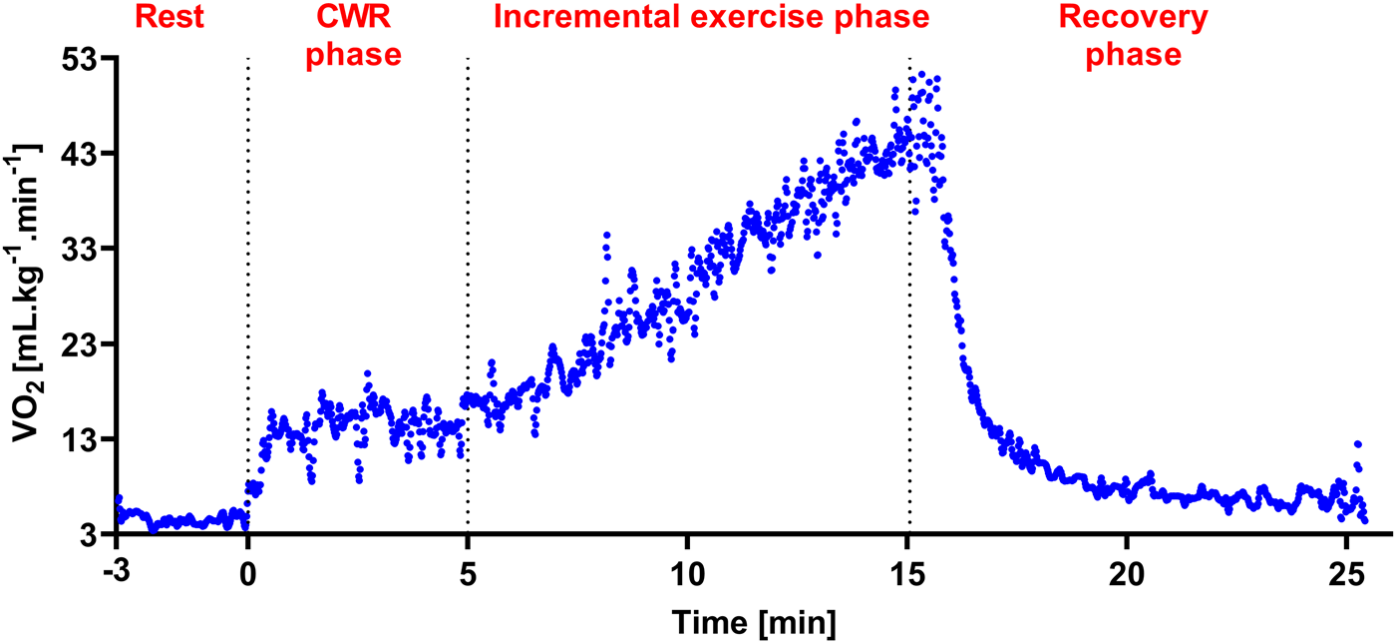
Continuous monitoring of ${\dot{V}}_{O_{2}}$ during the single CPET test for a single participant. Simultaneous determination of ${\dot{V}}_{O_{2}\mathrm{peak}}$ and ${\dot{V}}_{O_{2}}$ on/off‐kinetics can be performed in the identified phases. CWR, constant work rate; ${\dot{V}}_{O_{2}}$, oxygen consumption; ${\dot{V}}_{O_{2}\mathrm{peak}}$, peak oxygen consumption.

Figure 2 shows the continuous monitoring of oxygen consumption (${\dot{V}}_{O_{2}}$) throughout the single CPET test for a single participant. Simultaneous determination of ${\dot{V}}_{O_{2}\mathrm{peak}}$ and ${\dot{V}}_{O_{2}}$ on/off‐kinetics can be performed through the easily identifiable phases: CWR (on‐kinetics), incremental exercise (${\dot{V}}_{O_{2}\mathrm{peak}}$) and recovery (off‐kinetics).

The Shapiro–Wilk normality test and the Brown–Forsythe equal variance test reported no significant statistical differences (*P* > 0.05) for all main variables measured and predicted, such as ${\dot{V}}_{O_{2}\mathrm{peak}}$, steady‐state ${\dot{V}}_{O_{2}}$ (SS ${\dot{V}}_{O_{2}}$) at the CWR, the individuals’ 30% ${\dot{V}}_{O_{2}\mathrm{reserve}}$ measured, and the actual intensity, as a percentage (i.e., % ${\dot{V}}_{O_{2}\mathrm{reserve}}$), experienced by the participants during the CWR.

Descriptive statistics are represented in Table 2, where means ± SD are reported for the measured (M) and predicted (P)/calculated (C) main variables assessed along with the observed percentage difference (% Diff) or point difference (%P diff) between them. Regarding percentage difference (% Diff), generally, females had a slightly higher percentual difference than males for the prediction of both ${\dot{V}}_{O_{2}\mathrm{peak}}$ and the required SS ${\dot{V}}_{O_{2}}$ (∼10% vs. ∼9%; ∼11% vs. ∼7%, respectively), while for the combined group the difference was approximately ∼11% and ∼9%, respectively. Assessing the exercise intensity (%${\dot{V}}_{O_{2}\mathrm{reserve}}$) performed at the CWR phase, that is, the actual exercise intensity relative to ${\dot{V}}_{O_{2}\mathrm{reserve}}$ performed by the participants during the CWR, there was an overall mean difference of 0.2 percentage points from the aimed 30%${\dot{V}}_{O_{2}\mathrm{reserve}}$ exercise intensity in the combined group (Table 2).

**TABLE 2:** The measured and predicted ${\dot{V}}_{O_{2}\mathrm{peak}}$, SS ${\dot{V}}_{O_{2}}$ at CWR, ‘actual’ calculated individuals’ 30% ${\dot{V}}_{O_{2}\mathrm{reserve}}$, and the ‘actual’ calculated intensity as a percentage (%) of ${\dot{V}}_{O_{2}\mathrm{reserve}}$ experienced during the CWR for females, males and combined.

	Female (*n *= 9)	Male (*n *= 11)	Combined (*n *= 20)
(M) ${\dot{V}}_{O_{2}\mathrm{peak}}$ (mL kg^−1^ min^−1^)	28.8 ± 7.2	37.1 ± 7.8	35.4 ± 5.9
(P) ${\dot{V}}_{O_{2}\mathrm{peak}}$ (mL kg^−1^ min^−1^)	30.6 ± 3.9	39.3 ± 4.0	33.4 ± 8.5
% Diff. (%)	10.4 ± 23.8	9.4 ± 23.4	10.9 ± 23.2
(M) SS ${\dot{V}}_{O_{2}}$ (mL kg^−1^ min^−1^)	11.6 ± 1.4	13.7 ± 2.4	12.4 ± 2.8
(C) 30% ${\dot{V}}_{O_{2}\mathrm{reserve}}$ (mL kg^−1^ min^−1^)	10.7 ± 2.3	14.5 ± 1.4	13.2 ± 2.0
% Diff. (%)	11.4 ± 20.4	7.4 ± 12.0	9.3 ± 16.4
(C) % ${\dot{V}}_{O_{2}\mathrm{reserve}}$ at CWR (%)	29.2 ± 6.5	30.9 ± 9.9	30.2 ± 8.4
%P diff. (% points)	−1.0 ± 6.0	1.0 ± 9.0	0.2 ± 0.8

*Note*: Values are means ± SD. Percentage difference (%Diff.) between the measured variables and the predicted or calculated were assessed for the groups. Percentage point difference (%P. diff.), that is, difference from 30%, is reported for the calculated ${\dot{V}}_{O_{2}\mathrm{reserve}}$ observed during the CWR. Abbreviations: C, calculated; CWR, constant work rate; M, measured; P, predicted; SS, steady‐state.

### 3.1 Agreement between predicted and measured variables

Two‐sampled paired Student's *t*‐tests showed no statistically significant differences (*P* = 0.47) between predicted (P) and measured (M) ${\dot{V}}_{O_{2}\mathrm{peak}}$ (Table 3). Participants performed the CWR at an intensity of 30.2 ± 8.4% of their ${\dot{V}}_{O_{2}\mathrm{reserve}}$, which is not significantly different from the 30% initially set (one‐sample Student's *t*‐test, *P* = 0.93, Table 3).

**TABLE 3:** Mean difference analyses for the measured, predicted and calculated variables for the combined group (*n* = 20).

	Mean difference	Cohen's *d*	*P*‐value
(M) ${\dot{V}}_{O_{2}\mathrm{peak}}$ (mL kg^−1^ min^−1^)	0.82 (−1.8 to 1.5)	0.16 (−0.3 to 0.6)	0.47
(P) ${\dot{V}}_{O_{2}\mathrm{peak}}$ (mL kg^−1^ min^−1^)			
(C) % ${\dot{V}}_{O_{2}\mathrm{reserve}}$ at CWR (%)	0.18 (−3.7 to 4.1)	0.02 (−0.4 to 0.5)	0.93
Initially set 30% intensity (%)			

*Note*: Values are means (CI_95_); *n* = 20. *P*‐values determined by two‐sample paired Student's *t*‐test and one‐sample Student's *t*‐test (for the set 30% intensity). Abbreviations: C, calculated; CI_95_, 95% confidence interval band; CWR, constant work rate; M, measured; P, predicted.

Table 3 also reports the difference in the means (mean difference) and the size of the difference (i.e., Cohen's *d*) found in the tests along with the (CI_95_) for the appropriate comparisons. The mean difference was considered small between the predicted and measured ${\dot{V}}_{O_{2}\mathrm{peak}}$ and between percentage ${\dot{V}}_{O_{2}\mathrm{reserve}}$ at CWR and initially set at 30% intensity. The Cohen's *d* value between the predicted and measured ${\dot{V}}_{O_{2}\mathrm{peak}}$ was 0.16 and between the percentage ${\dot{V}}_{O_{2}\mathrm{reserve}}$ at CWR and the initially set at 30% intensity, was 0.02. These results endorse the idea that both variables overlapped reasonably well.

**FIGURE 3:**
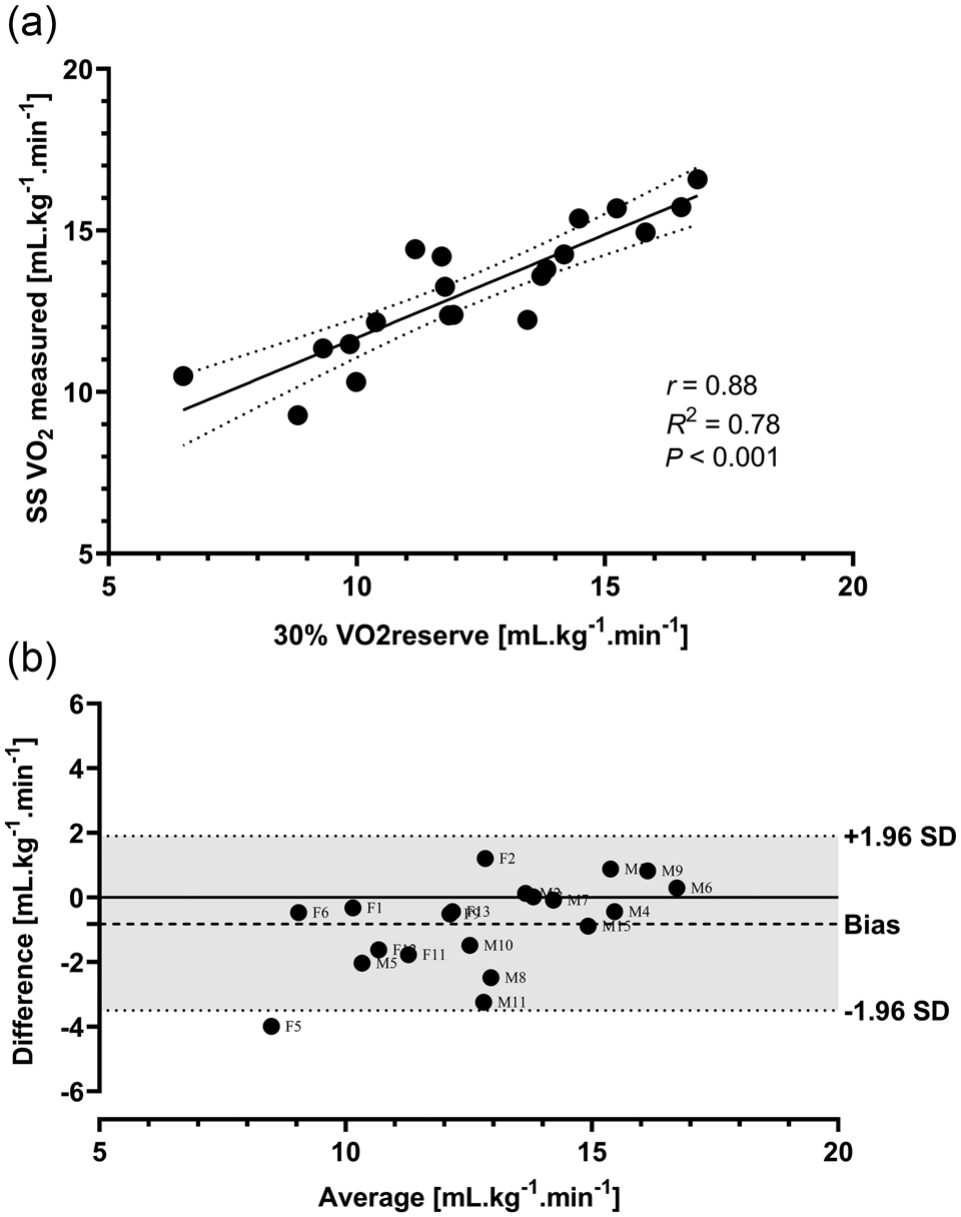
(a) Pearson's correlation between participants' 30% ${\dot{V}}_{O_{2}\mathrm{reserve}}$ and the SS ${\dot{V}}_{O_{2}}$ along with the linear regression (continuous line) and its corresponding 95% confidence interval (dotted lines). The coefficients of correlation (*r*) and determination (*R* ^2^) are also reported, where *P* < 0.05 is deemed significant. (b) Bland–Altman plot showing the agreement between the measured 30% ${\dot{V}}_{O_{2}\mathrm{reserve}}$ of the participants and the SS ${\dot{V}}_{O_{2}}$ measured during the CWR. The dotted lines represent the limits of agreement (CI_95_), the dashed line represents the mean bias, and the continuous line is set at zero. ‘F’ represents female and ‘M’ male participants. CWR, constant work rate; SS, steady state.

A strong correlation between the corresponding ${\dot{V}}_{O_{2}}$ value (mL kg^−1^ min^−1^) for the participants’ 30% ${\dot{V}}_{O_{2}\mathrm{reserve}}$ and the SS ${\dot{V}}_{O_{2}}$ measured during the CWR was found (*P* < 0.0001), with coefficient of correlation *r* = 0.88 (CI_95_: 0.72–0.95). In Figure 3a, the correlation between participants’ 30% ${\dot{V}}_{O_{2}\mathrm{reserve}}$ and the SS ${\dot{V}}_{O_{2}}$ is displayed along with the linear regression line (with CI_95_) and its coefficient of determination, *R* ^2^ = 0.78. The agreement between the SS ${\dot{V}}_{O_{2}}$ measured during the CWR and the participants’ 30% ${\dot{V}}_{O_{2}\mathrm{reserve}}$ measured/calculated was assessed using the Bland–Altman plot in Figure 3b. It shows a mean bias of −0.82 mL kg^−1^ min^−1^ (SD = 1.4), with limits of agreement from −3.5 to 1.9 mL kg^−1^ min^−1^.

**FIGURE 4:**
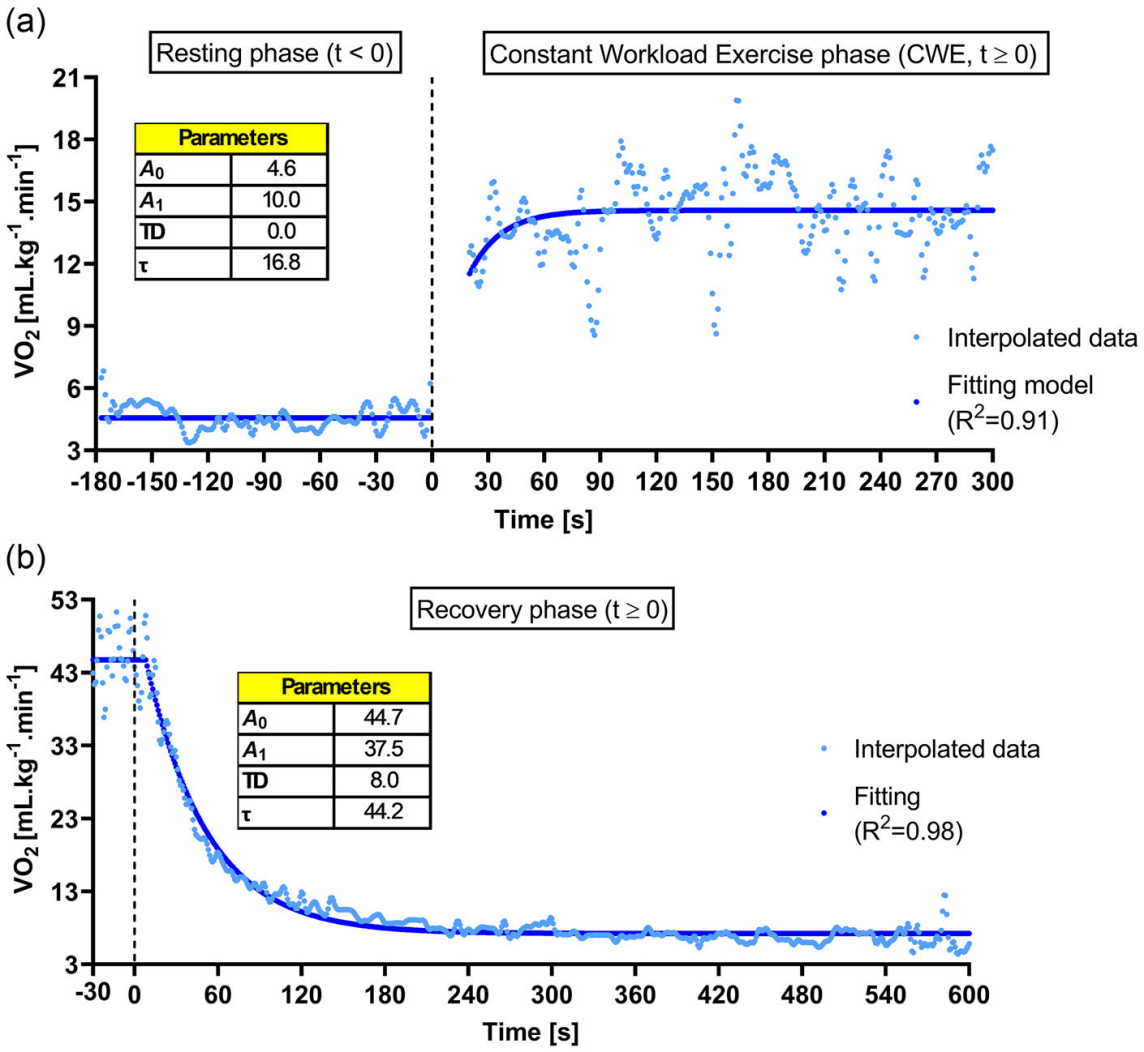
${\dot{V}}_{O_{2}}$ on/off‐kinetics of a representative individual. Removal of outliers (i.e., 3 SD) and second‐by‐second interpolation were performed before fitting. Analysis of ${\dot{V}}_{O_{2}}$ on‐kinetics (a) for the rest‐to‐exercise transition and ${\dot{V}}_{O_{2}}$ off‐kinetics (b) for the recovery phase of the protocol were performed using mono‐exponential fitting models. τ is the time constant of the exponential function, representing the time taken to reach 63% of the steady state or final value; TD represents the time delay taken to the exponential response start increase or decrease; *A*_0_ and *A*_1_ represent the baseline value (i.e., resting or ending value of ${\dot{V}}_{O_{2}}$) and the amplitude reached from baseline to steady‐state ${\dot{V}}_{O_{2}}$ (i.e., on‐kinetics) or from ${\dot{V}}_{O_{2}\mathrm{peak}}$ to baseline (i.e., off‐kinetics). CWR, constant work rate.

Analysis of ${\dot{V}}_{O_{2}}$ kinetics could be performed using the single protocol developed following common practices by fitting a mono‐exponential model to the breath‐by‐breath data collected (Hughson, 2009; Whipp & Rossiter, 2005). Figure 4 depicts the on/off ${\dot{V}}_{O_{2}}$ kinetics mono‐exponential analysis for a male participant. The collected data (blue dots) are presented after a 3 SD outlier removal process and second‐by‐second linear interpolation with the fitting model overlapped (continuous blue line). During the transition from rest to the CWR exercise phase of the proposed protocol (${\dot{V}}_{O_{2}}$ on‐kinetics), it was possible to determine the τ, TD, and amplitude (*A*_0_ and *A*_1_) (Figure 4a). Similarly, the parameters from exercise to recovery (${\dot{V}}_{O_{2}}$ off‐kinetics) were also determined (Figure 4b).

The mean ± SD of the main parameters that characterized the mono‐exponential fitting, including the mean response time (MRT = TD + τ), for the ${\dot{V}}_{O_{2}}$ on/off‐kinetics are described in Table 4 for the female, male and combined groups.

**TABLE 4:** Parameters obtained from the ${\dot{V}}_{O_{2}}$ kinetic analysis (on/off‐kinetics) performed with the female, male and combined groups.

	Female (*n *= 9)	Male (*n *= 11)	Combined (*n *= 20)
${\dot{V}}_{O_{2}}$ on‐kinetics			
*A*_0_ (mL kg^−1^ min^−1^)	3.7 ± 1.1	4.5 ± 0.6	4.1 ± 0.9
*A*_1_ (mL kg^−1^ min^−1^)	7.0 ± 1.5	9.3 ± 2.1	8.3 ± 2.2
TD (s)	3.1 ± 4.8	2.6 ± 5.6	2.8 ± 5.1
τ (s)	16.7 ± 6.4	28.4 ± 14.5	23.1 ± 12.8
MRT (s)	19.9 ± 6.8	31.0 ± 14.1	26.0 ± 12.5
${\dot{V}}_{O_{2}}$ off‐kinetics			
*A*_0_ (mL kg^−1^ min^−1^)	27.6 ± 6.4	36.0 ± 6.7	32.2 ± 7.8
*A*_1_ (mL kg^−1^ min^−1^)	22.3 ± 5.8	29.4 ± 6.0	26.2 ± 6.9
TD (s)	7.3 ± 5.8	3.0 ± 4.5	4.9 ± 5.6
τ (s)	49.6 ± 4.6	53.0 ± 6.2	51.5 ± 5.8
MRT (s)	57.0 ± 6.1	56.0 ± 7.5	56.5 ± 6.9

*Note*: Data are presented as means ± SD. Abbreviations: *A*_0_ and *A*_1_, amplitude; TD, time delay; τ, tau; MRT, mean response time.

## 4 DISCUSSION

To date, this is the first study to propose a systematically standardized single‐session CPET for the combined assessment of ${\dot{V}}_{O_{2}\mathrm{peak}}$ and ${\dot{V}}_{O_{2}}$ on/off‐kinetics. The small mean difference observed for the prediction of ${\dot{V}}_{O_{2}\mathrm{peak}}$ indicated that the Hansen–Wassermann equation was well‐suited for our population. Similarly, good agreement between the actual/calculated individuals’ 30% ${\dot{V}}_{O_{2}\mathrm{reserve}}$ and the measured aerobic power, SS ${\dot{V}}_{O_{2}}$, during the CWR exercise phase reflected a good match between the intended exercise intensity (i.e., 30% ${\dot{V}}_{O_{2}\mathrm{reserve}}$) and the actual intensity at the CWR phase, which was around 28.6% ${\dot{V}}_{O_{2}\mathrm{reserve}}$. The overall small Cohen's *d* values observed between predicted and measured ${\dot{V}}_{O_{2}\mathrm{peak}}$ and between SS ${\dot{V}}_{O_{2}}$ at the CWR and the participants’ measured 30% ${\dot{V}}_{O_{2}\mathrm{reserve}}$ also indicated the similarity and good overlap between the main variables. Additionally, the parameters characterizing the on/off‐kinetics (i.e., mono‐exponential models) can be determined using our approach. Therefore, our study demonstrates the feasibility of incorporating a combined assessment of ${\dot{V}}_{O_{2}\mathrm{peak}}$ and ${\dot{V}}_{O_{2}}$ on/off‐kinetics in a single‐session CPET, which can be expected to enhance participant recruitment and retention in clinical and exercise studies.

Longobardi et al. (2022) investigated the impact of severe COVID‐19 infection on ${\dot{V}}_{O_{2}\mathrm{peak}}$ and ${\dot{V}}_{O_{2}}$ on/off‐kinetics and cardiopulmonary function in a single‐session protocol to reduce the number of participants’ hospital visits. The exercise intensity at the CWR was subjectively determined based on participants’ maximum tolerable walking speed and rate of perceived exertion with a CWR phase lasting 3 min. However, this approach leads to participants exercising at different intensities during the CWR phase of the protocol, and the time may not be sufficient for all participants to reach an SS, particularly in populations with low ${\dot{V}}_{O_{2}\mathrm{peak}}$. The authors also introduced a 1 min active recovery after volitional exertion, which hinders a true off‐transient ${\dot{V}}_{O_{2}}$ kinetics analysis (Longobardi et al., 2022). Even very light exercise interferes with the transition from exercise to recovery, which compromises the interpretation of underlying regulatory mechanisms and information regarding fatigue, tolerance and physical fitness (Dupont et al., 2010). Therefore, in our study, we administered passive recovery immediately after the cessation of exercise in order to solely assess the ability of the body to recover from the stress without the use of positive feedback provided by light exercise.

To conduct a proper ${\dot{V}}_{O_{2}}$ kinetics analysis that yields insights into metabolic function/dysfunction, accurate control of exercise intensity, and time is critical. Light–moderate intensity must be met so that the individuals’ ${\dot{V}}_{O_{2}}$ responses have a steady‐state component during a CWR exercise, which is key to ensuring a physiologically stable condition to gain insights into the mechanisms underlying aerobic fitness (Hughson, 2009). Traditional approaches determine such intensity based on a percentage of the first ventilatory threshold (VT1) (George et al., 2018; Hughson & Morrissey, 1982; Murias et al., 2011b) or ${\dot{V}}_{O_{2}\mathrm{peak}}$/${\dot{V}}_{O_{2}\mathrm{reserve}}$ of the individuals (Garber, 2018; Wasserman et al., 2012) to obtain personalized and relative reference values of aerobic power. However, these approaches typically involve multiple days of testing (e.g., ${\dot{V}}_{O_{2}\mathrm{peak}}$/${\dot{V}}_{O_{2}\mathrm{reserve}}$) or at least several hours in the laboratory (e.g., VT1) to allow enough recovery time after the test in order to proceed and perform the CWR protocol (Beltrame et al., 2017; George et al., 2018; Murias et al., 2011b). All of these steps aim to ensure that participants perform the CWR exercise at a similar relative intensity, for example, everyone at a moderate intensity, to properly compare ${\dot{V}}_{O_{2}}$ kinetics. Nevertheless, these multiple steps or sessions simultaneously make the assessment of ${\dot{V}}_{O_{2}}$ kinetics cumbersome and, in many contexts, infeasible such as for hospitalized or vulnerable people (e.g., frail). Thus, establishing a systematic protocol for combining the assessment of ${\dot{V}}_{O_{2}\mathrm{peak}}$ and ${\dot{V}}_{O_{2}}$ on/off‐kinetics within a single session is not only desirable (e.g., financially, logistically) but crucial to achieving such a public, in which such types of studies are scarce.

To overcome this issue, in our current study, the intensity of the CWR exercise was estimated using well‐established predictive formulas from the literature (Garber, 2018; Wasserman et al., 2012). This intensity was calculated for each participant at 30% of their ${\dot{V}}_{O_{2}\mathrm{reserve}}$. The ${\dot{V}}_{O_{2}\mathrm{reserve}}$ is a relative method of prescribing exercise intensity, recommended over absolute methods (percentage ${\dot{V}}_{O_{2}\mathrm{peak}}$ or percentage maximum heart rate) (Garber, 2018), which provides a more accurate exercise intensity for the ${\dot{V}}_{O_{2}}$ on‐kinetics analysis. And indeed, the ${\dot{V}}_{O_{2}}$ kinetics analysis performed herein revealed that the relative intensity (% ${\dot{V}}_{O_{2}\mathrm{reserve}}$) at which the participants exercised (i.e., CWR phase) was around ∼30% of their ‘actual’ ${\dot{V}}_{O_{2}\mathrm{reserve}}$ measured post‐test (Table 3), which shows that the participants were exercising at a similar intensity regime, required for proper comparative ${\dot{V}}_{O_{2}}$ kinetics analysis (Whipp & Rossiter, 2005). The strong correlation and good agreement between the corresponding ${\dot{V}}_{O_{2}}$ value (mL kg^−1^ min^−1^) for the participants’ 30% ${\dot{V}}_{O_{2}\mathrm{reserve}}$ and the SS ${\dot{V}}_{O_{2}}$ measured during the CWR (Figure 3a, b) also endorse our protocol capacity to provide similar relative intensities for the CWR exercise phase. The magnitude of effect size between predicted and measured variables during the tests was small–medium size (Cohen, 1988), indicating considerable overlap in the data. Such results highlight the potential of our protocol to combine the assessment/measurement/evaluation of ${\dot{V}}_{O_{2}\mathrm{peak}}$ and ${\dot{V}}_{O_{2}}$ on/off‐kinetics in a single session.

The complexity of ${\dot{V}}_{O_{2}}$ kinetics is also influenced by time, specifically the time required for an individual's ${\dot{V}}_{O_{2}}$ response to reach SS during the CWR exercise and the time necessary to assess the recovery after exercise. Healthy young adults can reach SS in less than 3 min during moderate CWR exercise (Beltrame et al., 2017), while chronically ill older adults may take twice as long or more (Alexander et al., 2003; Arena et al., 2003; Murias & Paterson, 2015). Our proposed protocol takes these elements into account to perform ${\dot{V}}_{O_{2}}$ kinetics analysis (Figure 4). The time constant (τ) and mean response time (MRT) obtained with the analyses of on‐ and off‐kinetics described in Table 4 seem to be similar to what has been reported in previous studies for young individuals (Beltrame et al., 2017; George et al., 2018; Rossiter et al., 2002). In addition, young females have been shown to have apparently (no statistical test was performed) faster on‐kinetics than males, which has also been observed elsewhere (Beltrame et al., 2017). Studies evaluating ${\dot{V}}_{O_{2}}$ kinetics in older individuals have shown that τ can range from 30 to 60 s and with longer phase I (i.e., longer MRT) compared to young individuals (Murias & Paterson, 2015). Thus, considering that steady state is achieved within four time constants (4 × τ) (Hughson, 2009), the proposed 5 min‐long CWR exercise phase and the specified recovery time should be suitable for most individuals. However, it is important to recognize that critically ill individuals may require a longer time to reach SS and recover (Alexander et al., 2003). For instance, it has been reported that patients with heart failure may present τ ${\dot{V}}_{O_{2}}$ that ranges from 40 to 80 s, depending on whether the ejection fraction is preserved or not (Cipriano et al., 2024). Therefore, appropriate adaptations to this protocol might be necessary in such cases.

Indeed, the reliance on predictive equations (e.g., based on North American individuals) used and the proposed 5‐min CWR phase might raise generalizability concerns due to race, age or health status. The proposed protocol can be slightly adapted to address these critical points to achieve better results. For example, for healthy community‐dwelling older adults (60–80 years), predictive equations for ${\dot{V}}_{O_{2}\mathrm{peak}}$ have been developed specifically for this population (Šagát et al., 2023; Sewell et al., 2024), and could be used instead of the Hansen–Wasserman equations. Similarly, race could be addressed using predictive equations that target the ethnicity of the sample under study (de Souza e Silva et al., 2018; Puente‐Maestú et al., 2021). However, predictive equations for more frail populations, for instance, those recently discharged from the intensive care unit (ICU), can be more challenging and require special caution. For example, Benington et al. (2012) identified that their sample of ICU survivors (6 weeks post‐discharge), had achieved only 56% of their predicted ${\dot{V}}_{O_{2}\mathrm{peak}}$ using a cycle ergometer. Mart et al. (2022) and Longobardi et al. (2022) also reported lower ${\dot{V}}_{O_{2}\mathrm{peak}}$ values for their sample of COVID‐19 ICU survivors (3–5 months post‐discharge) compared to predictive equations, but much higher than the ones reported by Benington et al. (2012) of around 80% and 83%, respectively. Understandably, the deleterious impact on aerobic capacity and aerobic power in such populations will likely vary broadly depending on several factors such as the reason for hospitalization (e.g., COVID‐19, sepsis, heart failure), the need for mechanical ventilation and the time from discharge. Therefore, when using standardized predictive equations for such vulnerable populations with unique health conditions, an implementation of a correction factor of 0.6, 0.7 or 0.8, for example, based on direct measures found in the literature, might be necessary. This approach will allow for the development of predictive equations specifically tailored to the target population, addressing their unique health limitations and providing more accurate estimates of their maximum aerobic capacity and aerobic power.

The length of the CWR could also be explored for cases where extremely slow kinetics is expected. However, the length of the CWR phase might compromise the incremental test that follows. In fact, we have acknowledged that further validation studies are needed not only to fully elucidate this point but also to compare and validate the measurements obtained with the proposed single‐session protocol against standard procedures for ${\dot{V}}_{O_{2}}$ kinetics and ${\dot{V}}_{O_{2}\mathrm{peak}}$ analyses (e.g., multiple stepwise transitions or Bruce protocol). Adaptations to the proposed protocol will likely be necessary in practice to tailor the characteristics of the population under assessment. Thus, the flexibility of the proposed protocol stands out as a key strength. Making it suitable for diverse populations (e.g., according to age or health status), would enhance the applicability of exercise testing in both research and clinical settings. The possibility of implementing a complete exercise testing protocol, particularly for more vulnerable/frail populations that includes ${\dot{V}}_{O_{2}}$ kinetics analysis, for example, is of extreme importance. For instance, Longobardi et al. (2022) have recognized the usefulness of such an approach to acquire crucial insights into long‐term metabolic dysfunctions (e.g., oxidative phosphorylation and fatigue‐related metabolites), usually attributed to survivors of severe COVID‐19 (Longobardi et al., 2022; Wang et al., 2022).

### 4.1 Limitations

As previously mentioned, the inherent reliance on predicted ${\dot{V}}_{O_{2}\mathrm{peak}}$ for a specific population will inevitably limit the generalizability of our findings. Although it has been widely implemented for decades with similar predictive accuracy observed in this study (Puente‐Maestú et al., 2021), the Hansen–Wasserman equations are primarily derived from middle‐aged and sedentary individuals in North America. Nevertheless, a key strength of the protocol is flexibility. The option to use predictive equations developed for a specific population such as older adults or frailer individuals (Benington et al., 2012; Mart et al., 2022), as detailed in this section, can minimize errors and make predictions for the population under investigation.

Another point to consider is that multiple trials (generally three bouts) of CWR exercise tests are usually recommended when the aim is to perform ${\dot{V}}_{O_{2}}$ kinetics analysis to maximize the signal‐to‐noise ratio and guarantee proper fitting quality (Koga et al., 2005; McNulty & Robergs, 2019). However, incorporating multiple trials of CWR exercise in this single protocol may impact the incremental portion of the maximal CPET, prohibiting this approach. Nevertheless, despite multiple trials being considered the recommended statistical approach to achieve better fitting results, researchers have opted for a single test session due to logistical factors and resource limitations (Arena et al., 2003; Chatterjee et al., 2013; Longobardi et al., 2022). Our current study showed that we were able to derive an appropriate mono‐exponential curve using a single bout of CWR. We acknowledge that a comparative analysis with traditional methods would enhance the validity of the results. However, we did not perform a comparative analysis, as this study was designed as a feasibility study rather than a validation study. Its primary aim was to identify potential challenges and provide preliminary data to justify future validation studies.

Lastly, it is important to note that the current study was not designed to validate the measured values derived from our protocol. We understand that the data collected herein must be compared with reference values obtained using standard procedures, for example, multiple stepwise transitions for ${\dot{V}}_{O_{2}}$ kinetics analysis and Bruce protocol for ${\dot{V}}_{O_{2}\mathrm{peak}}$. Also, the transition from ${\dot{V}}_{O_{2}\mathrm{peak}}$ to rest is a different off‐kinetic metric from a transition from CWR; in this regard, the interpretation of these novel time constants has yet to be determined. Thus, this current study aims to shed light on possible systematic methods on which exercise testing can be adapted and, at the same time, provide useful insights into cardiopulmonary health. This novel protocol will consider the reality of clinical and research settings with time constraints and participants’ adherence challenges.

Despite these limitations, the flexible framework of our protocol enables the application of suitable predictive equations tailored to the population of interest. It also permits easy adjustments to the target CWR and duration of incremental phases, accommodating diverse populations. Moreover, the length of recovery can be adapted to allow proper recovery time for off‐kinetics analysis. Therefore, various populations can derive benefits from this standardization due to its simplicity and customization capacities, making it an excellent option when addressing challenges regarding recruitment and retention rates, as well as the affordability of research and clinical exercise testing.

### 4.2 Conclusions

In conclusion, we have developed a systematic and standardized single‐session CPET for the combined assessment of ${\dot{V}}_{O_{2}\mathrm{peak}}$ and ${\dot{V}}_{O_{2}}$ on/off‐kinetics. Designing a single protocol that integrates ${\dot{V}}_{O_{2}\mathrm{peak}}$ and ${\dot{V}}_{O_{2}}$ on/off‐kinetics will address challenges associated with patient recruitment and retention in research and provide further detailed information for clinical CPET use. A simplified and single protocol offers specific advantages, particularly for individuals at higher risk or those living with disabilities. Future studies should focus on validating this protocol to ensure its intra‐participant reliability and generalizability in other populations, for example, with varying levels of fitness and health conditions.

## AUTHOR CONTRIBUTIONS

All experiments were conducted in the Cardiorespiratory and Physiology of Exercise Research Laboratory in the Active Living Centre, room 240, at the University of Manitoba. All authors contributed to the study's conception and design. Material preparation, data collection, and analysis were performed by Jefferson L. Santana, Till Enzner, Britney Blunderfield and Rodrigo Villar. The first draft of the manuscript was written by Jefferson L. Santana, Till Enzner and Rodrigo Villar, and all authors commented on previous versions of the manuscript. All authors read and approved the final manuscript and agree to be accountable for all aspects of the work in ensuring that questions related to the accuracy or integrity of any part of the work are appropriately investigated and resolved. We also confirm that all persons designated as authors qualify for authorship, and all those who qualify for authorship are listed.

## CONFLICT OF INTEREST

None declared.

## Data Availability

Data can be obtained upon request from the corresponding author.
